# Use of Naltrexone–Bupropion in Persons With Overweight/Obesity and Symptoms of Depression: A Pooled Analysis

**DOI:** 10.1002/oby.70183

**Published:** 2026-03-31

**Authors:** Jena Shaw Tronieri, Robert F. Kushner, Caroline M. Apovian, Angela Fitch, Christopher Still, Thomas A. Wadden

**Affiliations:** ^1^ Department of Psychiatry, Center for Weight and Eating Disorders Perelman School of Medicine at the University of Pennsylvania Philadelphia Pennsylvania USA; ^2^ Department of Medicine Northwestern University Feinberg School of Medicine Chicago Illinois USA; ^3^ Brigham and Women's Hospital Center for Weight Management and Wellness Boston Massachusetts USA; ^4^ Knownwell Needham Massachusetts USA; ^5^ Geisinger Commonwealth School of Medicine Danville Pennsylvania USA

**Keywords:** depression, pharmacologic therapy, weight management

## Abstract

**Objective:**

The present study examined the safety and efficacy of the fixed‐dose, extended‐release combination of naltrexone and bupropion (NB‐ER) in individuals with overweight/obesity and mild to moderate symptoms of depression.

**Methods:**

Data were pooled from four double‐blind, placebo‐controlled trials. Participants with baseline Inventory of Depressive Symptomatology—Self‐Report scores ≥ 14 suggestive of mild or greater depressive symptoms were included (*N* = 511). Primary outcomes were 56‐week percent weight loss and changes in depression, as well as safety data for psychiatric adverse events (PAEs), depressive symptom increases, and suicidal ideation. Multiple imputation was applied, and outcomes were compared in the intention‐to‐treat population.

**Results:**

In participants with symptoms of depression, 56‐week weight loss was significantly greater with NB‐ER (5.7% ± 0.6%) than with placebo (2.7% ± 0.8%; *p* = 0.003). Participants experienced clinically meaningful improvements in depression of −7.1 ± 0.4 with NB‐ER and −6.7 ± 0.5 with placebo, with no significant differences between the groups. NB‐ER and placebo did not differ in safety signals including PAE occurrence (27.5% NB‐ER vs. 22.1% placebo), depressive symptom increases (9.5% NB‐ER vs. 8.8% placebo), or suicidal ideation (1.8% NB‐ER vs. 2.0% placebo).

**Conclusions:**

NB‐ER appears to be both safe and effective for weight loss when offered to patients with mild to moderate symptoms of depression.

## Study Importance

- What is already known?
  ○ A significant minority of patients with overweight/obesity also present with depression.
  ○ Treatment recommendations for co‐occurring obesity and depression are limited because few randomized trials have evaluated safety and efficacy in this population.
- What does this study add?
  ○ In this pooled analysis of individuals with mild to moderate baseline symptoms of depression participating in four randomized controlled trials, naltrexone–bupropion ER (NB‐ER) induced a mean 5.7% reduction in baseline body weight at 56 weeks that was significantly larger than the 2.7% loss of participants treated with placebo.
  ○ Both groups experienced significant reductions in symptoms of depression, and NB‐ER did not increase the likelihood of having increases in depressive symptoms, psychiatric adverse events, or suicidal ideation.
- How might these results change the direction of research or the focus of clinical practice?
  ○ Treatment providers can be more confident that NB‐ER is both safe and effective for weight loss when prescribed to patients presenting with overweight/obesity and mild to moderate symptoms of depression.
  ○ Future randomized trials should investigate the effects of NB‐ER in patients who have been formally diagnosed with major depressive disorder.

## 1 Introduction

Obesity and depression frequently co‐occur [1], which has been attributed to shared pathophysiology, behavioral patterns, and psychosocial determinants [2, 3]. An analysis of 2005–2010 NHANES data reported that 43% of US adults with depression have obesity, as compared to 33% of adults without depression [4]. Obesity and depression are independently associated with poorer quality of life [3], increased risk of other chronic medical conditions such as cardiovascular disease and diabetes [5, 6], higher health care costs [7, 8], and higher all‐cause mortality [9, 10]. Effective medication treatments have been developed for each, but there is minimal guidance on which approaches to select for patients presenting with both conditions [2]. Special consideration is necessary given that many psychotropic medications can cause weight gain [11, 12], and some may be less effective in the presence of obesity [3]. Additionally, some centrally acting obesity medications have raised safety concerns due to elevated depression risk [13, 14].

The fixed‐dose, extended‐release combination of naltrexone and bupropion (NB‐ER) was approved by the Food and Drug Administration for chronic weight management in 2014. Bupropion is a dopamine and norepinephrine reuptake inhibitor indicated for the treatment of depression, seasonal affective disorder, and smoking cessation. Its role in weight loss has been attributed to stimulation of pro‐opiomelanocortin neurons in the hypothalamus that decrease food intake and increase energy expenditure, as well as to dopamine's role in food reward [15]. Naltrexone is an opioid receptor antagonist commonly used in the treatment of substance use disorders. Opioid receptors play a role in food palatability, and naltrexone potentially reduces reactivity to food cues [16]. In combination, the two components have a synergistic effect due to naltrexone's ability to block autoinhibitory reductions in the anorexigenic effects of bupropion [15].

In a pooled analysis of 3362 participants from the four Phase 3 clinical trials in the “Contrave Obesity Research” (COR) program, NB‐ER produced a 7% loss of baseline body weight at 56 weeks, compared to a 2.3% loss with placebo [17]. A separate pooled analysis found that the percentage of participants reporting depression‐related psychiatric adverse events (PAEs) was lower with NB‐ER (0.8%) than with placebo (1.5%), but total PAE incidence was higher (22% vs. 15.5%, respectively) [18]. The NB‐ER group also had a small mean increase in depressive symptoms compared to a small decrease with placebo. However, mean depression scores were low at baseline, and this difference was primarily attributed to changes in item ratings that were not unique to depression [18].

The 2022 American Gastroenterological Association guidelines [19], as well as several experts [2, 20], have recommended NB‐ER for patients with co‐occurring obesity and depression due to the bupropion component's role in the treatment of depressive disorders. However, other guidelines have cautioned against its use for this population without further direct evaluation [21]. Evidence of the efficacy and safety of NB‐ER in patients with depressive symptoms is limited. One uncontrolled pilot study found that patients with major depression and obesity lost 5.3% of initial body weight and had > 50% mean reductions in symptoms of depression with NB‐ER after 24 weeks [22]. However, without a comparison group, it is difficult to interpret these effects, particularly given the high rates of placebo response in many depression studies [22]. In a secondary analysis of data from the NB‐ER cardiovascular outcome trial [23], participants who were taking antidepressant medications during the trial did not have a significantly larger weight loss with NB‐ER than with placebo at Year 2, further highlighting the need for placebo‐controlled comparisons to establish the medication's efficacy in patients with depression.

The present study compared the efficacy and safety of NB‐ER to placebo in patients with symptoms of depression. Data from 511 participants who reported mild to moderate depressive symptoms at baseline were pooled from four, 56‐week, randomized trials that compared naltrexone ER (16 or 32 mg/day) + bupropion ER (360 mg/day) to placebo in participants with overweight/obesity. The co‐primary efficacy endpoints were (1) percent change in body weight and (2) changes in symptoms of depression, both as measured from baseline to Week 56. Safety analyses focused on treatment‐emergent concerns relevant to depression, including PAEs, elevated depressive symptoms, and suicidal ideation.

## 2 Methods

### 2.1 Study Designs

This was a post hoc, pooled analysis of data from four Phase 3 clinical trials: COR‐I (NCT00532779) [24]; COR‐II (NCT00567255) [25]; COR‐BMOD (NCT00456521) [26]; and COR‐Diabetes (NCT00474630) [27]. All were 56‐week, multicenter, double‐blind, randomized controlled trials conducted between 2007 and 2009. Study protocols were approved by an institutional review board at each site, and all participants provided written informed consent. Detailed descriptions of the studies' designs, eligibility criteria, treatments, and outcomes have been published previously [24, 25, 26, 27].

### 2.2 Participants

#### 2.2.1 Eligibility for Parent Study Participation

Across studies, participation was open to adults free from serious medical conditions (e.g., significant cardiovascular disease). In COR‐I, COR‐II, and COR‐BMOD, participants were aged 18–65 years without diabetes and had a body mass index (BMI) of 30–45 kg/m^2^ or a BMI of 27–45 kg/m^2^ with controlled hypertension and/or dyslipidemia. In COR‐Diabetes, participants were 18–70 years of age with type 2 diabetes (HbA1c between 7% and 10% and fasting blood glucose < 270 mg/mL) and a BMI of 27–45 kg/m^2^. Allowable medications are described in the Extended Methods in online Supporting Information.

All studies excluded individuals based on the following psychiatric conditions: current severe major depressive disorder or active suicidal ideation; current personality disorder; past 6‐month suicide attempt or psychiatric hospitalization; past‐year drug or alcohol abuse; and lifetime history of severe psychiatric illnesses including bipolar disorder, psychosis, bulimia, or anorexia nervosa. Current mood stability was further assessed using the Inventory of Depressive Symptomatology—Self‐Report (IDS‐SR) [28], and only individuals with total scores < 30 and item scores < 2 for sadness, irritability, anxiety/tension, and suicidality at screening were eligible. Individuals taking psychotropic medications (with exception of short‐term insomnia treatments) within the past 6 months also were excluded, as were participants who had taken bupropion or naltrexone within 12 months.

#### 2.2.2 Eligibility for the Present Substudy

Participants were included in this study if they had a total IDS‐SR score ≥ 14 at baseline. This threshold was selected because scores 0–13 are thought to be indicative of not having depression.

### 2.3 Treatments

In each study, participants were randomized to receive NB‐ER or placebo. Most participants who received active medication were assigned a dose of 32 mg/day naltrexone + 360 mg/day bupropion. Exceptions were as follows: in COR‐I, half received 16 mg/day naltrexone + 360 mg/day bupropion (and half received 32 mg/day) [24]; and in COR‐II, participants who did not lose ≥ 5% of their baseline weight between Weeks 28 and 44 were re‐randomized to either continue the 32 mg/day formulation or escalate to 48 mg/day naltrexone (still with 360 mg/day bupropion) [25]. All doses were delivered in two oral tablets taken twice daily, and all studies included a 3– to 4‐week dose escalation period.

All study participants received counseling to follow a hypocaloric diet (500 kcal/day energy deficit) and increase physical activity, along with advice on lifestyle modification. In COR‐I, COR‐II, and COR‐Diabetes, ancillary counseling was provided individually at baseline and at four additional visits spread throughout the trial period. COR‐BMOD instead provided an intensive behavior modification program delivered in groups of 10–20 participants. Groups met for 90 min once weekly for 16 weeks then every other week for 12 weeks (a total of 28 sessions) and received a full curriculum on lifestyle modification strategies [26].

### 2.4 Outcomes

Across all four trials, participants were assessed at baseline and every 4 weeks to evaluate the following outcomes.

#### 2.4.1 Body Weight

Weight was measured at each visit on calibrated scales. The primary weight outcome was percent change in baseline body weight at Week 56, and secondary outcomes were achievement of a categorical weight loss ≥ 5%, ≥ 10%, and ≥ 15% at that time.

#### 2.4.2 Symptoms of Depression

The primary depression outcome was 56‐week change in the IDS‐SR total score [28]. Participants rated, on a 0–3 scale, the intensity with which they had experienced 30 symptoms of depression within the past week. The total score ranges from 0 to 84. Scores of 14–25 are considered to indicate mild depressive symptoms, 26–38 moderate symptoms, and 39 or above severe or very severe symptoms [29]. Secondary outcomes were having no (or minimal) depressive symptoms at Week 56, defined as a score < 14 at that time, and symptom response, defined as a ≥ 50% reduction from baseline to Week 56. Safety outcomes were having a clinically significant increase in depressive symptoms ≥ 10 points above baseline at one or more post‐baseline assessment [29] and post‐baseline scores ≥ 2 on the suicidality item, which indicate thoughts of suicide or death several times a week or more.

#### 2.4.3 Psychiatric Adverse Events

Treatment‐emergent adverse events were collected throughout each trial. Recorded PAEs were updated to preferred terms and categories using the Medical Dictionary for Regulatory Activities (MedDRA), version 28.0 [30]. Adverse events were selected for analysis if they could be hierarchically classified within the “psychiatric disorder” system organ class, including adverse event terms with dual membership in an alternate class.

### 2.5 Statistical Analyses

Comparisons between NB‐ER and placebo in primary and secondary outcomes were conducted in the intention‐to‐treat (ITT) population using SPSS Statistics v.28.0.1.1, except as indicated. Missing values for body weight and IDS‐SR scores were first estimated with multiple imputation (MI) [31, 32]. Following MI, the co‐primary outcomes were evaluated using repeated measures linear mixed‐effects models including all available assessment data. Participants' achievement of categorical weight loss and depression targets was calculated using MI data, and group proportions were compared using chi‐square tests. R (v.4.4.3) package *miceadds* [33] was used to pool results using Rubin's rules [34]. Additional model details can be found in the Extended Methods in online Supporting Information.

Because this was a post hoc study, an a priori power analysis was not conducted and corrections for multiple tests were not applied. For the co‐primary outcomes, we conducted sensitivity analyses including: modified ITT (mITT) in the safety population, which comprised participants who were administered ≥ 1 medication dose and provided at least one post‐baseline measurement; mITT in the original primary efficacy population that completed at least one of those assessments within 1 day of taking a dose of study medication; and completers. Conclusions reported in the text regarding between‐group differences were consistent across all models.

Safety outcomes were analyzed in the safety population using only available data. Chi‐square or Fisher's exact tests (if expected frequency was < 5) were used to compare the proportions of participants who reported at one or more post‐baseline visit: (1) any PAE; (2) a PAE within MedDRA categories relevant to depression (depressed mood disorders and disturbances; mood disorders and disturbances; suicidal and self‐injurious behaviors); (3) a PAE within any other category with incidence ≥ 5% in either group; (4) an IDS‐SR increase ≥ 10 points above baseline; and (5) IDS‐SR suicidality. Similar analyses were used to explore between‐group differences in the specific PAEs that appeared most commonly.

Lastly, using logistic regression, we explored demographic and baseline characteristics (age; race/ethnicity; sex; body weight; depression), 8‐week changes in body weight and depressive symptoms, IDS‐SR increases ≥ 4 points, and PAEs as predictors of study attrition.

## 3 Results

### 3.1 Participants' Baseline Characteristics

A total of 511 participants with baseline symptoms of depression were selected, 351 of whom had been assigned to NB‐ER and 160 to placebo. Most NB‐ER participants received the 32 mg/day dose of naltrexone (*n* = 295; 84%), and 56 received the 16 mg/day dose. Table S1 shows the breakdown by parent study and NB‐ER dosage.

The selected participants' baseline demographic characteristics (Table 1) resembled those of the parent trials. The majority were female (87%) and identified as White (81%) and non‐Hispanic (90%). Participants had a mean body weight of 99.3 kg (SD = 15.5) and mean depression score of 18.2 (SD = 4.4; range 14–41). The treatment groups did not differ significantly in baseline depressive symptoms.

**TABLE 1:** Participant characteristics at baseline.

Characteristic	Total (*N* = 511)	Naltrexone + bupropion ER (*N* = 351)	Placebo (*N* = 160)
Sex (female), *n* (%)	447 (87.5%)	307 (87.5%)	20 (87.5%)
Race, *n* (%)			
White	414 (81.0%)	281 (80.1%)	133 (83.1%)
Black	72 (14.1%)	50 (14.2%)	22 (13.8%)
Multiracial or other	25 (4.9%)	20 (5.7%)	5 (3.1%)
Ethnicity (Hispanic), *n* (%)	52 (10.2%)	31 (8.8%)	21 (13.1%)
Age (years)	47.2 ± 11.4	47.2 ± 11.3	47.2 ± 11.4
Weight (kg)	99.3 ± 15.5	100.0 ± 16.3	97.9 ± 13.6
BMI (kg/m^2^)	36.4 ± 4.2	36.6 ± 4.3	36.1 ± 4.2
Symptoms of depression (IDS‐SR)	18.2 ± 4.4	18.3 ± 4.6	17.8 ± 4.1

*Note*: Values shown are *N* (%) or mean ± SD.

Abbreviations: ER, extended release; IDS‐SR, Inventory of Depressive Symptomatology—Self‐Report.

### 3.2 Retention

Of these 511 participants, 480 (93.9%; 331 assigned to NB‐ER and 149 to placebo) were included in the safety population. At Week 56, 267 participants (52.3%; 188 assigned to NB‐ER and 79 to placebo) completed the IDS‐SR and 264 (51.7%) had a measured body weight. Out of 14 total post‐baseline assessments, the average participant completed 8.9 measurements for both depression (SD = 5.6) and body weight (SD = 5.8), with no differences between the treatment groups (*p* = 0.733 and *p* = 0.975, respectively).

### 3.3 Weight Loss

**FIGURE 1:**
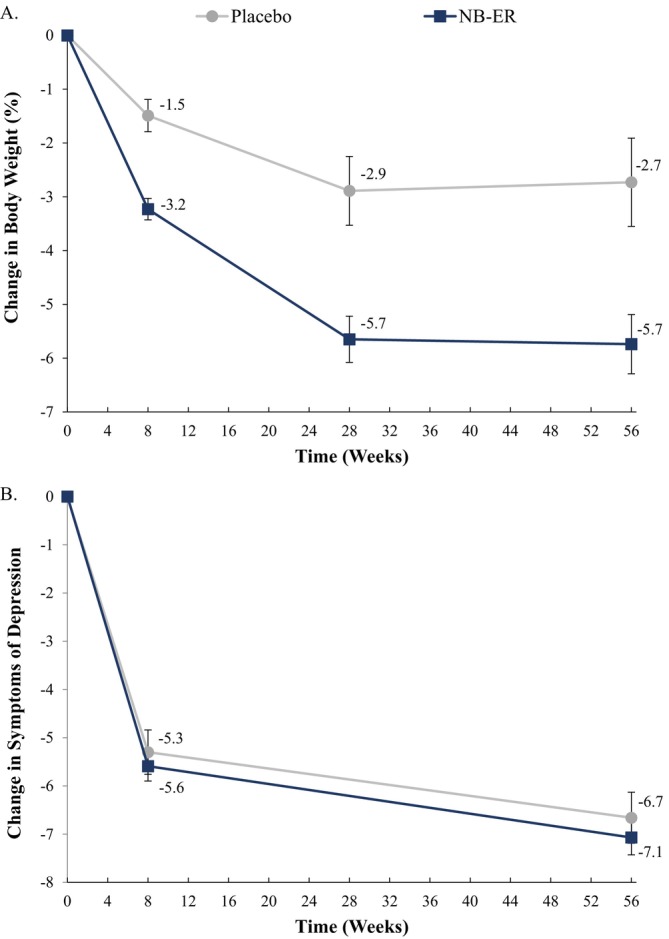
Changes in body weight and symptoms of depression across the 56‐week treatment period. Values are estimated mean changes (± standard errors [SE]) from randomization to Week 56 in the intention‐to‐treat (ITT) population (*N* = 511; 351 assigned to NB‐ER and 160 to placebo) derived from linear mixed models applied to 35 multiply imputed datasets. Mean estimates and their SE were then pooled using Rubin's rules. Available data from all 14 post‐baseline assessments occurring every 4 weeks throughout the treatment period contributed to the models (mean = 8.9 assessments per person for both outcomes). Breakpoints were determined for each outcome using model fit statistics (e.g., −2 log likelihood). (A) Percent changes in baseline body weight. (B) Changes in symptoms of depression as measured by the total score on the Inventory of Depressive Symptomatology—Self‐Report. NB‐ER, naltrexone + bupropion extended release. [Color figure can be viewed at wileyonlinelibrary.com]

From baseline to Week 56, participants assigned to NB‐ER had a significantly greater mean (±SE) percent reduction in body weight of 5.7% ± 0.6% (95% CI: 4.6%–6.9%) compared to the 2.7% ± 0.8% (95% CI: 1.1%–4.4%) reduction in the placebo group (estimated MD: 3.0% ± 1.0%, 95% CI: 1.0%–5.0%, *p* = 0.003, Figure 1A). Participants who completed the Week 56 assessment on drug had lost 8.1% ± 0.6% with NB‐ER and 3.5% ± 0.9% with placebo (estimated MD: 4.6% ± 1.1%, *p* < 0.001). Estimates from sensitivity analyses can be found in Table S2.

**FIGURE 2:**
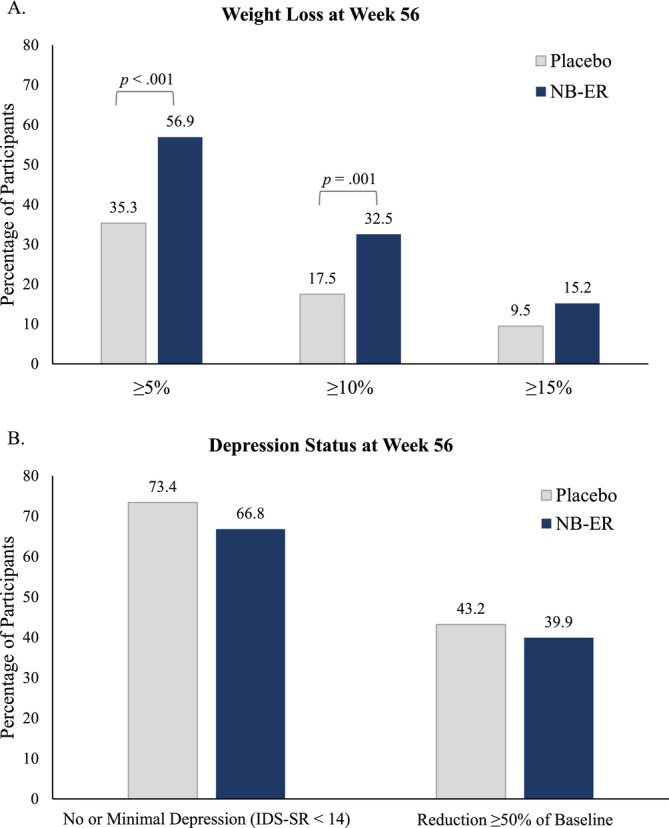
Categorical weight loss and depression outcomes at Week 56. Proportions are based on the intention‐to‐treat sample (*N* = 511; 351 assigned to NB‐ER and 160 to placebo) with missing body weight and depression symptom scores estimated from 35 multiply imputed datasets. (A) Percentage of participants assigned to each treatment group who lost ≥ 5%, ≥ 10%, and ≥ 15% of baseline weight at Week 56. The categories are not mutually exclusive. For example, the percentage who lost ≥ 5% includes those who lost ≥ 10% and ≥ 15%. (B) Percentage of participants in each treatment group who achieved categorical depression outcomes of: (1) having no or minimal symptoms of depression (IDS‐SR < 14) and (2) reductions ≥ 50% of baseline depressive symptoms at Week 56. IDS‐SR, Inventory of Depressive Symptomatology—Self‐Report; NB‐ER, naltrexone + bupropion extended release. [Color figure can be viewed at wileyonlinelibrary.com]

At Week 56, an estimated 56.9% of NB‐ER‐treated participants lost ≥ 5% of baseline weight, 32.5% lost ≥ 10%, and 15.2% lost ≥ 15% (Figure 2A). Among participants in the placebo group, an estimated 35.3% lost ≥ 5% of baseline weight, 17.5% lost ≥ 10%, and 9.5% lost ≥ 15%. Significantly more NB‐ER participants met the 5% and 10% weight loss targets than did those who received placebo (*p*s ≤ 0.001), but differences in achievement of a ≥ 15% loss were not statistically significant (*p* = 0.105).

### 3.4 Symptoms of Depression

Both groups experienced 56‐week improvements in symptoms of depression of −7.1 ± 0.4 with NB‐ER and −6.7 ± 0.5 with placebo (Figure 1B). These improvements were medium to large in size (Cohen's *d* = −0.84 for NB‐ER and *d* = −0.54 for placebo) and did not differ significantly (*p* = 0.538, Table S2). Results for assessment completers who were on drug were similar (−7.1 ± 0.5 with NB‐ER and −7.1 ± 0.8 with placebo). Additionally, there were no differences between the treatment groups in the percentage of participants who achieved categorical targets of having no or minimal depression (*p* = 0.219) or symptom improvement ≥ 50% from baseline (*p* = 0.506) at Week 56 (Figure 2B).

We explored whether depression outcomes differed in COR‐BMOD due to the greater intensity of interventionist contact provided during that study in comparison to the other three trials. In COR‐BMOD, symptoms of depression decreased by 6.3 ± 0.9 from baseline to Week 56 in participants treated with NB‐ER (*n* = 44) and by 8.5 ± 1.3 with placebo (*n* = 22, *p* = 0.174). In the remaining three trials combined, 56‐week reductions were 7.2 ± 0.4 with NB‐ER and 6.4 ± 0.6 with placebo (*p* = 0.416).

### 3.5 Safety

#### 3.5.1 Psychiatric Adverse Events

PAEs occurred in 27.5% of participants treated with NB‐ER and 22.1% of those treated with placebo, with no significant difference between the groups in overall incidence (Table 2). The treatment groups did not differ significantly in the proportion of participants who experienced PAEs in the depressed mood disorder (3.9% and 2.7%, respectively) or mood disorder category (5.1% and 6.7%, respectively). The most common mood‐related PAEs were irritability, experienced by 4.2% and 4.7% of participants in the NB‐ER and placebo groups, respectively (*p* = 0.816), and depression, experienced by 2.1% and 1.3%, respectively (*p* = 0.727). No participant in either group experienced suicidal/self‐injurious behavior PAEs.

**TABLE 2:** Psychiatric adverse events in the safety population.

Category—Psychiatric adverse event, *n* (%)	Naltrexone + bupropion ER (*N* = 331)	Placebo (*N* = 149)	*p*
Any psychiatric adverse event	91 (27.5%)	33 (22.1%)	0.216
Anxiety disorders and symptoms	22 (6.6%)	8 (5.4%)	0.593
Anxiety	20 (6.0%)	6 (4.0%)	
Panic attack	2 (0.6%)	0 (0%)	
Stress	0 (0%)	2 (1.3%)	
Tension	1 (0.3%)	1 (0.7%)	
Acute stress disorder	1 (0.3%)	0 (0%)	
Depressed mood disorders and disturbances	13 (3.9%)	4 (2.7%)	0.495
Depression	7 (2.1%)	2 (1.3%)	
Depressed mood	5 (1.5%)	2 (1.3%)	
Persistent depressive disorder	1 (0.3%)	0 (0%)	
Mood disorders and disturbances	17 (5.1%)	10 (6.7%)	0.488
Irritability	14 (4.2%)	7 (4.7%)	
Affect lability	1 (0.3%)	1 (0.7%)	
Anger	1 (0.3%)	0 (0%)	
Apathy	0 (0%)	1 (0.7%)	
Emotional distress	1 (0.3%)	0 (0%)	
Mood altered	0 (0%)	1 (0.7%)	
Mood swings	0 (0%)	1 (0.7%)	
Sleep disorders and disturbances	49 (14.8%)	12 (8.1%)	0.040
Insomnia	32 (9.7%)	6 (4.0%)	
Somnolence	9 (2.7%)	0 (0%)	
Sleep disorder	4 (1.2%)	2 (1.3%)	
Abnormal dreams	5 (1.5%)	0 (0%)	
Initial insomnia	2 (0.6%)	2 (1.3%)	
Middle insomnia	1 (0.3%)	2 (1.3%)	
Nightmare	1 (0.3%)	0 (0%)	
Suicidal and self‐injurious behaviors NEC	0 (0%)	0 (0%)	—

*Note*: The safety population included the 480 participants who were exposed to a study drug and returned for at least one post‐baseline assessment. Psychiatric adverse events were classified using the Medical Dictionary for Regulatory Activities (MedDRA) v.28.0. “Category” is used to refer to the MedDRA high‐level group term and “psychiatric adverse event” to the specific preferred term for each event. Categories were selected for presentation if directly relevant to patients with depression or if they had an incidence rate ≥ 5% in either group. Within those categories, all specific events that occurred in this sample are presented. The “Any psychiatric adverse event” row shows the total number of participants who experienced ≥ 1 psychiatric event, including events within the categories presented in the table and those classified under categories that did not meet the presentation criteria. There were no significant differences between the treatment groups in any psychiatric adverse event category that did not meet the prespecified ≥ 5% presentation threshold.

Abbreviations: ER, extended release; NEC, not elsewhere classified.

Sleep disorders/disturbances were the most common PAE subtype in both groups. Significantly more NB‐ER participants (14.8%) reported sleep‐related PAEs as compared to placebo (8.1%). This difference was driven, in part, by differences in the percentage of participants who reported insomnia, which was higher with NB‐ER (9.7%) than with placebo (4.0%, *p* = 0.034), though this effect was attenuated when all insomnia‐related PAE terms were grouped together (10.6% vs. 6.7%, *p* = 0.179). Although somnolence and abnormal dreams also appeared more frequently with NB‐ER, those differences were not statistically significant in this sample (*p* = 0.063 and *p* = 0.331, respectively).

The only other PAE category with incidence ≥ 5% in either group was anxiety disorders and symptoms, which were experienced by a similar percentage of participants treated with NB‐ER (6.6%) and placebo (5.4%). The groups also did not differ in the percentage who reported anxiety, specifically (*p* = 0.367).

#### 3.5.2 Increases in Symptoms of Depression

In the 475 participants who completed at least one post‐baseline IDS‐SR measurement, 9.5% (*n* = 31) of those treated with NB‐ER and 8.8% (*n* = 13) of those treated with placebo had clinically meaningful increases in depressive symptoms at any post‐baseline assessment (*p* = 0.833). Of these, the vast majority had only one (77.3%) or two (15.9%) assessments with scores in this range. Only 1.8% (*n* = 6) of participants treated with NB‐ER and 2.0% (*n* = 3) of those treated with placebo had suicidality scores ≥ 2 at any post‐baseline visit (*p* = 0.890).

### 3.6 Predictors of Attrition

Sex, baseline weight, baseline depressive symptoms, 8‐week changes in depressive symptoms, and experiencing any PAEs or sleep disorder/disturbance PAEs during the trial were not significantly associated with study completion at Week 56 (Table 3). In the final multivariable model, having a non‐White/Hispanic race/ethnicity (OR = 0.50, 95% CI: 0.30–0.82, *p* = 0.006) and depressive symptoms ≥ 4 points above baseline at Week 8 (OR = 0.30, 95% CI: 0.13–0.71, *p* = 0.006) independently predicted lower odds of completing the study, and larger 8‐week weight losses predicted greater odds of completion (OR = 0.91, 95% CI: 0.85–0.99, *p* = 0.019). Higher age was associated with study completion in univariate analysis (Table 3) but not when controlling for these variables (OR = 1.02, 95% CI: 0.995–1.04, *p* = 0.135). No predictors interacted significantly with treatment condition, indicating that effects were similar in magnitude in both groups.

**TABLE 3:** Individual analyses examining predictors of study completion at Week 56.

Predictor variable	*N*	Variable		Interaction with treatment group	
		Odds ratio (95% CI)	*p*	Odds ratio (95% CI)	*p*
Age (years)	511	1.02 (1.004–1.05)	0.018	0.99 (0.96–1.03)	0.600
Sex (0: male vs. 1: female)	511	0.62 (0.32–1.20)	0.159	2.15 (0.67–6.90)	0.198
Race (0: White vs. 1: non‐White)	511	0.52 (0.32–0.86)	0.010	0.50 (0.20–1.26)	0.140
Baseline body weight (kg)	511	1.002 (0.99–1.02)	0.752	1.01 (0.98–1.03)	0.641
Baseline depressive symptoms (IDS‐SR)	511	0.98 (0.93–1.02)	0.288	0.98 (0.90–1.08)	0.712
Week 8 weight change (% change in baseline body weight)^a^	412	0.86 (0.78–0.95)	0.002	1.04 (0.89–1.22)	0.612
Week 8 change in depressive symptoms (IDS‐SR)^b^	407	0.97 (0.93–1.01)	0.145	1.05 (0.98–1.13)	0.148
Week 8 IDS‐SR ≥ 4 points above baseline (0: no vs. 1: yes)^c^	407	0.26 (0.10–0.65)	0.004	1.92 (0.25–14.94)	0.534
Any psychiatric adverse event (0: no vs. 1: yes)	480	0.78 (0.47–1.30)	0.345	1.37 (0.53–3.53)	0.522
Sleep disturbance adverse event (0 no vs. 1: yes)	480	1.10 (0.58–2.08)	0.774	0.60 (0.15–2.43)	0.478

*Note*: Analyses were conducted separately for each predictor variable and its interaction with treatment condition, controlling for study. Study completion in this subsample was similar to the parent trials. At Week 56, 264 participants (51.7%) completed the study with a measured body weight. The dependent variable, completion at Week 56, was scored such that a value of 1 represented visit completion, and odds ratios (ORs) significantly greater than 1 indicated higher odds of completing the study. For continuous changes in body weight and depressive symptoms at Week 8, more negative change scores indicated greater improvement (and more positive scores indicated deterioration). Therefore, ORs significantly greater than 1 signify that smaller improvements/larger deteriorations predicted greater odds of completing the study, and ORs significantly less than 1 indicate that greater improvements predicted greater odds of completing the study.

^a^ Results were similar when excluding COR‐BMOD, which had higher 8‐week and 56‐week weight losses as well as higher study completion (*N* = 352, weight change OR = 0.87 [0.79–0.96], *p* = 0.006; weight change × treatment OR = 1.03 [0.87–1.23], *p* = 0.695).

^b^ Continuous change in IDS‐SR at Week 8 also did not significantly predict study completion when substituted for categorical change ≥ 4 points in the multivariable analysis (OR = 0.99 [0.62–1.62], *p* = 0.70).

^c^ A total of 28 participants (6.9%) had increases in IDS‐SR depressive symptoms ≥ 4 at Week 8. Of these, only 11 (39.3%) completed the Week 56 visit as compared to 65.4% of the 379 individuals who did not experience this early symptom elevation.

## 4 Discussion

This study's principal findings were that NB‐ER, relative to placebo, improved weight loss in participants with mild to moderate symptoms of depression and did not raise any new safety concerns when used in this population. The mean percentage weight loss of participants treated with NB‐ER (5.7%) was more than double that of those assigned to placebo (2.7%) after 56 weeks of treatment. Approximately 57% of NB‐ER‐treated participants lost ≥ 5% of baseline body weight, a common threshold for clinically meaningful weight loss, whereas only 35% achieved this target when treated with placebo. Participants who received NB‐ER were also more likely to achieve losses ≥ 10% of initial weight.

Both treatment groups experienced clinically significant reductions in depressive symptoms of approximately 7 points on the IDS‐SR from baseline to Week 56. The groups did not differ significantly in symptom improvement. This pattern differed from that of the parent trials, in which pooled change from baseline was minimal and differed significantly between groups (+0.1 for NB‐ER and −0.5 for placebo) due to more participants who received NB‐ER endorsing items related to appetite change and other somatic symptoms [18].

The lack of significant differences between groups in reductions in depressive symptoms is not altogether surprising in view of other intervention studies that have targeted both depression and weight loss. For example, a pilot study in patients with obesity and major depression found no differences in 46‐week symptom improvement in participants who received intensive lifestyle intervention for weight loss, cognitive behavioral therapy (CBT) for depression, or a combined lifestyle + CBT intervention [35]. Similarly, a larger trial comparing lifestyle intervention alone to lifestyle + CBT for depression in patients with both conditions found no group differences in 52‐week changes in depression [36].

Because behavioral weight loss interventions can produce improvements in depression that are similar in magnitude to treatments designed to target that condition [35, 36], we explored whether depression outcomes differed in COR‐BMOD, which was the only Phase 3 NB‐ER trial to provide participants with intensive lifestyle treatment. We did find that between‐group differences in depression reduction were slightly larger when excluding COR‐BMOD, but they still were not statistically significant. Surprisingly, in COR‐BMOD itself, mean reductions in depressive symptoms were non‐significantly larger with placebo than with NB‐ER. We caution over‐interpretation of this result given the small number of participants drawn from that trial (*n* = 66). In another small study, participants with binge eating disorder who had mean scores on the Beck Depression Inventory–II in the mild range at baseline had non‐significantly larger improvements in depression when they received behavioral weight loss plus NB‐ER (11.6 points) than combined with placebo (7.1 points) [37]. Thus, whether the effects of NB‐ER versus placebo on depression differ in the context of behavioral intervention appears to warrant further investigation in a larger sample.

Safety data suggested that participants with symptoms of depression did not experience PAEs, clinically significant increases in depressive symptoms, or suicidal ideation more frequently with NB‐ER than with placebo. There were no between‐group differences in the frequency of PAEs related to depression, mood, or anxiety disorders. In contrast, sleep disorders/disturbances were reported by significantly more participants treated with NB‐ER (14.8%) than with placebo (8.1%). Insomnia was the most commonly reported PAE and occurred more frequently in NB‐ER‐treated participants. Somnolence and abnormal dreams were also numerically more common with NB‐ER, but comparisons between groups did not reach statistical significance. In addition to the strong overall relationship between sleep disturbances and depression [38, 39], a number of antidepressant medications, including bupropion, have been associated with higher rates of short‐term insomnia in placebo‐controlled studies [40].

A pooled analysis of PAEs in the full sample of participants from these same four Phase 3 trials (plus one Phase 2 trial) also found that a higher percentage of participants treated with NB‐ER reported sleep disorder PAEs, while frequencies in the depression and anxiety categories did not differ between treatment groups (though they did find differences in some subtopics within those categories) [18]. Interestingly, PAE frequencies in the present subset of participants were similar to those of the overall pooled sample. For example, anxiety disorder PAEs were reported by 6.6% of participants with baseline depressive symptoms treated with NB‐ER and 5.4% of those treated with placebo, as well as by 6.1% and 4.4%, respectively, in the overall participant pool [18]. Together, these findings suggest that having mild to moderate symptoms of depression did not put participants at increased risk of having PAEs with NB‐ER.

Clinically meaningful increases in depressive symptoms ≥ 10 points occurred in less than 10% of participants in either group, and most of those participants' scores were only elevated at 1–2 of up to 14 possible assessments. Only 1.8% of participants treated with NB‐ER and 2.0% of those treated with placebo endorsed having thoughts of suicide or death at any post‐baseline assessment. No suicidal/self‐injurious behavior PAEs were recorded in this subsample. These results provide additional safety reassurance given that NB‐ER carries the Boxed Warning applied to antidepressant medications regarding the possibility of emerging suicidal thoughts and behaviors, particularly in children, adolescents, and young adults. Our findings indicate that NB‐ER is not likely to increase risk when used in adults with mild to moderate symptoms of depression. However, given that less than 4% of the sample was aged < 25 years, we cannot draw conclusions for that high‐risk age group.

Early increases in depressive symptoms of ≥ 4 points at Week 8 predicted attrition in both treatment groups. Early depression exacerbations appear to be a stronger predictor of dropout than either lower continuous early symptom improvement or greater baseline symptoms for patients with mild to moderate depression and should be considered a potential target for clinical intervention in this population. Similar to treatment studies in the general population, both slower early weight loss and non‐White race/ethnicity also predicted attrition in both groups [41], and strategies to improve outcomes for these populations should continue to be investigated.

A primary limitation of this study was that participants were not assessed by a clinician to determine whether they met diagnostic criteria for a depressive disorder. Thus, we may have included participants whose depressive symptoms were transient and likely to remit on their own or whose symptoms were attributable to a condition other than depression, obscuring potential treatment effects. Additionally, individuals with IDS‐SR symptom scores ≥ 30 (the lower end of the moderate range) or requiring psychotropic medication at screening were excluded. We cannot determine whether the present results would generalize to individuals with more severe depressive symptoms or to those taking antidepressant medications. Further, a majority of participants in all four trials were female, non‐Hispanic, and White, and results may not generalize to other populations. We also note that only 52% of participants completed the full 56‐week trial. Because outcomes were captured frequently throughout the trials, we could apply robust methods of missing data estimation. Sensitivity analyses also demonstrated that results remained consistent across different exposure populations. However, we could only examine the occurrence of safety outcomes such as PAEs within the completed visits (mean of nine per participant).

## 5 Conclusion

Our findings suggest that NB‐ER is both safe and effective for weight loss in adults with mild to moderate symptoms of depression. These outcomes provide preliminary support for clinical guidelines recommending NB‐ER for patients with overweight/obesity and depression. A larger placebo‐controlled trial in patients diagnosed with major depression and comparisons to other obesity medications are needed to fully establish evidence‐based guidelines and ensure that these findings are generalizable.

## Funding

This work was supported by Currax Pharmaceuticals LLC.

## Conflicts of Interest

J.S.T. reports receiving an investigator‐initiated grant on behalf of the University of Pennsylvania from Novo Nordisk and receiving consulting fees from Currax Pharmaceuticals LLC. R.F.K. reports serving on advisory boards or consulting for honoraria from Altimmune; Antag, AstraZenica, Boehringer Ingelheim; Currax Pharmaceuticals LLC; Eli Lilly; Novo Nordisk, Regeneron; Structure; and Weight Watchers. C.M.A. reports receiving grants paid to her institution from GI Dynamics and the Patient‐Centered Outcomes Research Institute; serving as the treasurer for the World Obesity Federation; and receiving consulting fees from AbbVie; Altimmune; Arrowhead Pharmaceuticals; BioAge Labs; Biolinq; Caribou Biosciences; CinFina Pharma, Covidien; Cowen and Company; Currax Pharmaceuticals LLC; EPG Communication Holdings; Form Health; Keros Therapeutics; Lilly; L‐Nutra; Mediflix; NeuroBo Pharmaceuticals; Neurocrine Biosciences; NodThera; Novo Nordisk; Nutrisystem; OptumRx; PainScript; Palatin Technologies; Pursuit By You; Redesign Health; ReShape Lifesciences; Riverview School; Roman Health Ventures; Scholar Rock; Terns; Verily Life Sciences; Veru; Vida Health; Wave Life Sciences; WondrHealth; Xeno Biosciences; and Zyversa Therapeutics. A.F. reports work on advisory boards for Currax Pharmaceuticals LLC, Eli Lilly, Ms.Medicine, Novo Nordisk, Rhythm Pharmaceuticals, SideKick Health, Seca, and VIVUS. C.S. reports serving on advisory boards or consulting for honoraria for Boehringer Ingelheim; Currax Pharmaceuticals LLC; Lilly; Novo Nordisk, Regeneron, and Weight Watchers, serving on speakers bureaus for Novo Nordisk and Lilly, and receiving research funds from Regeneron and Rhythm Pharmaceuticals. T.A.W. reports serving on advisory boards for Weight Watchers Inc. and receiving grants on behalf of the University of Pennsylvania from Eli Lilly and Novo Nordisk.

## Supporting information


**Data S1:** oby70183‐sup‐0001‐supinfo.docx.

## Data Availability

Deidentified participant data can be made available to investigators for research purposes on a case‐by‐case basis after the time of this publication upon written request to the corresponding author.
