# Supplementary material for: Use of Naltrexone–Bupropion in Persons With Overweight/Obesity and Symptoms of Depression: A Pooled Analysis
Source: Obesity (Silver Spring). 2026 Mar 31;34(5):1010–9. doi: 10.1002/oby.70183 (PMC13116053; doi:10.1002/oby.70183)
Supplement: Supplementary file 1 — Data S1: oby70183‐sup‐0001‐supinfo.docx. [file OBY-34-1010-s001.docx]

**Use of Naltrexone-Bupropion in Persons with Overweight/Obesity**

**and Symptoms of Depression: A Pooled Analysis**

**Supplementary Appendix**

Jena Shaw Tronieri, Robert F. Kushner, Caroline M. Apovian, Angela Fitch

Christopher Still, Thomas A. Wadden

**Extended Methods**

**Additional Eligibility Information**

**Other medication usage in the parent trials**. All four trials permitted usage of medications for hypertension (with exception of alpha-adrenergic blockers and clonidine) and dyslipidemia, as long as dose stable for 6 weeks prior to randomization. All trials excluded use of drugs or dietary supplements that significantly affect body weight (including anti-obesity medications) and of investigational drugs or devices within the past month. Participants in COR-Diabetes could not be using injectable hypoglycemic medications or inhaled insulin. They could be taking oral hypoglycemic medications if dose stable for at least 3 months.

As noted in the text, individuals taking psychotropic medications within the past 6 months also were excluded, with exception of short-term insomnia treatments. Allowable medications were up to 2 mg/day lorazepam or equivalent dose of a benzodiazepine or hypnotic agent, and short-term was generally defined as usage of 3 months or less.

**IDS-SR scores at baseline**. Although only individuals with IDS-SR scores < 30 at screening were eligible for the parent studies, individuals who met this criterion remained eligible even if their baseline score was above this threshold if approved by their site investigator as not having developed severe depression. Therefore a small minority of participants (9 NB-ER-treated and 3 placebo-treated) had baseline scores ≥30. When excluding these 11 individuals, mean changes and between-group differences in change in IDS-SR depressive symptoms and body weight were similar to those reported in the text.

**Statistical Methods**

Comparisons between NB-ER and placebo in primary and secondary outcomes were conducted in the intention-to-treat (ITT) population using SPSS Statistics v.28.0.1.1, except as indicated. Missing values for body weight and IDS-SR total scores were estimated with multiple imputation (MI) using chained equations. All 4-week assessments of each dependent variable were included in the imputation model, and study, treatment group, initial BMI, and demographic characteristics (age, sex, race, ethnicity) were entered as additional predictors. MI relies on the missing at random assumption, and 35 iterations were determined to be sufficient based on the fraction of missing data (γ = 0.31 to 0.39).^31,32^

Following MI, continuous changes in the co-primary outcomes were evaluated using repeated measures linear mixed-effects models including all available assessment data. Treatment group was entered as a between-subjects factor and time (week) was a within-subjects factor. The models’ shapes (piecewise) and variance-covariance structures (unstructured) were selected based on the -2 Log Likelihood and Akaike’s Information Criterion (AIC). Least squared means were used to compare 56-week total changes at a two-tailed α level of 0.05. Participants’ achievement of categorical weight loss and depression targets was calculated using MI data and group proportions were compared using chi square tests (α=0.05). R (v.4.4.3) package *miceadds*^33^ was used to pool continuous mean and mean difference estimates and their standard errors using Rubin’s rules,^34^ as well as to pool test statistics.

Controlling for baseline weight, IDS-SR (where appropriate), and demographic characteristics yielded similar results to the models presented in the main text. We also explored whether baseline depression moderated the treatment effects by comparing participants with baseline IDS-SR scores ≥18 to those with scores of 14-17. Although we did find that participants with higher baseline depression had larger improvements in depressive symptoms at week 56 (-10.4 with NB-ER and -10.0 with placebo) than those with lower scores (-4.4 and -4.3, respectively), baseline depression was not a significant moderator of the difference between the treatment groups.

For the co-primary outcomes, we conducted sensitivity analyses including 1) ITT using linear mixed models without multiple imputation, which estimate missing data via maximum likelihood; modified ITT (mITT) in the safety population, which included participants who were administered at least one medication dose and provided at least one post-baseline measurement 2) using MI with linear mixed models as describe above and 3) using LOCF; 4) mITT using LOCF in the original primary efficacy population of participants who also had completed ≥1 post-baseline assessment within 1 day of taking a dose of study medication; 5) completers; and 6) completers who had taken ≥1 dose of study medication within 1 day of the week 56 assessment. Results of these analyses can be found in Supplemental Table 2.

We also conducted post-hoc analyses of predictors of study attrition at week 56, including: baseline demographic and patient characteristics (age; race/ethnicity categorized as White vs non-White including Hispanic ethnicity; sex; body weight); early changes in body weight and IDS-SR depressive symptoms at week 8; and experiencing a psychiatric adverse event (PAE). Because only a minority of participants experienced increases in depressive symptom scores at any assessment, we believed that the continuous analysis could obscure whether early exacerbations in depression predicted dropout (as opposed to smaller vs larger improvements). We therefore also conducted a categorical analysis in which having an early increase in depressive symptoms was defined as an IDS-SR score ≥4 units above baseline at week 8. (Our primary safety analyses had defined increases in depression as ≥10 IDS-SR units; however, the number of participants experiencing elevations of that magnitude within the first 8 weeks was not sufficient to allow for meaningful analysis.) The threshold of ≥4 units was designed to capture increases in depressive symptoms of approximately 1 sample standard deviation or greater. Additionally, we specifically examined experiencing a Sleep Disorder & Disturbance PAE as a predictor of attrition, because this was the only PAE category reported more frequently by participants treated with NB-ER than placebo.

Analyses examining predictors of week 56 completion (1) vs attrition (0) were conducted using logistic regressions that included the relevant predictors and their interactions with treatment condition, controlling for study (attrition was lower in COR-BMOD). Analyses were first conducted for each independent variable separately, then variables potentially related to attrition at *p* < 0.10 were combined in a single multivariable logistic regression analysis to determine independent effects. We removed interaction terms from the final multivariable model because no significant interactions with treatment group were identified in either individual or preliminary multivariable analysis. Analyses of baseline predictors included all participants (*N* = 511), analyses of PAEs included the safety population for whom this data was available (*N* = 480), and analyses of early treatment changes were limited to participants who provided a week 8 measurement (*N* = 412 for body weight and *N* = 407 for IDS-SR depressive symptoms).

**Relationship Between Weight Loss and Changes in Symptoms of Depression**

We also explored the relationship between weight loss and changes in symptoms of depression. Using MI data, there was a small correlation between percent weight loss and IDS-SR change at week 56 (*r*=0.12, *p*=0.052). In the completer sample, there was no relationship between changes in these outcomes (*r*=0.03, *p*=0.62, *N*=250). Treatment condition did not moderate this relationship.

Supplemental Table S1. Selection of participants with IDS-SR ≥14 at baseline by parent study.

| Study | Total Randomized *N* | Total with  IDS-SR ≥ 14  *N* (% of total) | Total Randomized to NB-ER  *N* | NB-ER with IDS-SR ≥14  *N* (% of total) | Total Randomized to Placebo  *N* | Placebo  with IDS-SR ≥ 14  *N* (% of total) |
| --- | --- | --- | --- | --- | --- | --- |
| COR-I | 1742 | 185 (10.6%) | 1161 | 130 (11.2%) | 581 | 55 (9.5%) |
| COR-II | 1496 | 187 (12.5%) | 1001 | 127 (12.7%) | 495 | 60 (12.1%) |
| COR-Diabetes | 505 | 73 (14.5%) | 335 | 50 (14.9%) | 170 | 23 (13.5%) |
| COR-BMOD | 793 | 66 (8.3%) | 591 | 44 (7.4%) | 202 | 22 (10.9%) |
| Total | 4536 | 511 (11.3%) | 3088 | 351 (11.4%) | 1448 | 160 (11.0%) |

Participants who were assigned to NB-ER received naltrexone 32mg/d + bupropion 360 mg/d with the following exceptions: In COR-I, 583 of the total randomized were assigned to naltrexone 32mg/d and 578 to naltrexone 16mg/d, both plus bupropion 360 mg/d. Of those with baseline IDS-SR ≥14 selected from that study, 74 had received naltrexone 32mg/d and 56 received 16 mg/d. In COR-II, 244 of the 1001 NB-ER participants were re-randomized to either continue naltrexone 32mg/d or increase to 48mg/d for the remainder of the trial after having a loss ≤5% of initial weight between week 28-44. In the total study sample, dose was increased for 120 participants, and in the selected subsample, dose was increased for 21 participants. In exploratory analyses, results did not vary by naltrexone dose. Thus data are presented for the combined NB-ER group as planned.

*Note:* NB-ER = naltrexone + bupropion extended release; IDS-SR = Inventory of Depressive Symptomatology – Self-Report; COR = Contrave Obesity Research; BMOD = Behavior Modification.

Supplemental Table S2. Estimated mean percent reduction in body weight and changes in symptoms of depression from baseline to week 56 using different methods of missing data estimation and in different analytic populations

| **Variable** | **Naltrexone + Bupropion - ER** | **Placebo** | **Mean Difference**  **(95% CI)** | ***p* value** |
| --- | --- | --- | --- | --- |
| **Change in body weight (%)** | |  |  |  |
| LMM after MI, ITT | -5.7 ± 0.6 | -2.7 ± 0.8 | 3.0 ± 1.0 (1.0 to 5.0) | 0.003 |
| LMM alone, ITT/mITT^a^ | -5.8 ± 0.5 | -2.6 ± 0.7 | 3.3 ± 0.9 (1.5 to 5.1) | <0.001 |
| LMM after MI, mITT | -6.2 ± 0.5 | -2.8 ± 0.8 | 3.3 ± 1.0 (1.4 to 5.2) | <0.001 |
| LOCF, mITT | -5.4 ± 0.4 | -2.5 ± 0.6 | 2.9 ± 0.7 (1.6 to 4.2) | <0.001 |
| LOCF, mITT on drug | -6.4 ± 0.4 | -2.5 ± 0.6 | 3.9 ± 0.7 (2.5 to 5.3) | <0.001 |
| Completers | -7.8 ± 0.6 | -3.6 ± 0.9 | 4.1 ± 1.0 (2.1 to 6.2) | <0.001 |
| Completers on drug | -8.1 ± 0.6 | -3.5 ± 0.9 | 4.6 ± 1.1 (2.5 to 6.8) | <0.001 |
| **Change in symptoms of depression (IDS-SR)** | |  |  |  |
| LMM after MI, ITT | -7.1 ± 0.4 | -6.7 ± 0.5 | 0.4 ± 0.7 (-0.9 to 1.7) | 0.538 |
| LMM alone, ITT/mITT^a^ | -6.6 ± 0.4 | -6.4 ± 0.6 | 0.1 ± 0.8 (-1.4 to 1.7) | 0.855 |
| LMM after MI, mITT | -7.0 ± 0.4 | -6.6 ± 0.5 | 0.4 ± 0.7 (-0.9 to 1.7) | 0.537 |
| LOCF, mITT | -5.4 ± 0.4 | -5.7 ± 0.6 | -0.3 ± 0.7 (-1.8 to 1.1) | 0.648 |
| LOCF, on drug mITT | -5.8 ± 0.4 | -6.4 ± 0.6 | -0.6 ± 0.8 (-2.1 to 0.9) | 0.455 |
| Completers | -7.0 ± 0.5 | -7.3 ± 0.8 | -0.4 ± 0.7 (-2.1 to 1.4) | 0.688 |
| Completers on drug | -7.0 ± 0.5 | -7.1 ± 0.8 | -0.1 ± 0.9 (-1.9 to 1.7) | 0.914 |

Data are means or estimated marginal means (± *SE*). ITT analyses included data from all 511 participants (*n*=351 NB-ER and *n*=160 placebo) who had IDS-SR total scores ≥14 at baseline. mITT analyses included data from only the 480 participants (479 for IDS-SR; *n*=331 NB-ER and *n*=149 placebo) who were administered at least one medication dose and provided at least one post-baseline measurement (safety population), and “mITT on drug” analyses were restricted to participants who had also taken ≥1 dose of study medication within 1 day of at least one post-baseline assessment (body weight *n*=413, *n*=269 NB-ER and *n*=144 placebo; IDS-SR *n*=432, *n*=289 NB-ER and *n*=143 placebo). Completer analyses included only participants who provided a measurement at week 56 (body weight *n*=264, *n*=182 NB-ER and *n*=82 placebo; IDS-SR *n*=267, *n*=188 NB-ER and *n*=79 placebo), and “completer on drug” analyses were restricted to those who had also taken ≥1 dose of study medication within 1 day of the week 56 assessment (body weight *n*=242, *n*=165 NB-ER and *n*=77 placebo; IDS-SR *n*=259, *n*=182 NB-ER and *n*=77 placebo). Statistical conclusions regarding between-group differences were consistent across all samples and methods of missing data handling. The primary analyses reported in the text were ITT analyses using LMM after MI. The main outcome papers for all four studies used LOCF in the “mITT on drug” sample for their primary efficacy analyses.

^a^ Although all 511 participants were included in the “LMM alone” analyses, participant with no post-baseline measurement contribute minimally to slope estimation, and mITT results for change over time are nearly identical for these models.

*Note:* ITT = intention-to-treat; mITT = modified intention-to-treat; LMM = linear mixed model; MI = multiple imputation; LOCF = last observation carried forward; NB-ER = naltrexone and bupropion, extended-release; IDS-SR = Inventory of Depressive Symptomatology Self-Report.
